# Immunotherapy Approaches in HPV-Associated Head and Neck Cancer

**DOI:** 10.3390/cancers13235889

**Published:** 2021-11-23

**Authors:** Ricklie Julian, Malvi Savani, Julie E. Bauman

**Affiliations:** Department of Medicine, Division of Hematology/Oncology, University of Arizona, Tucson, AZ 85724, USA; rickliej@arizona.edu (R.J.); mbsavani@arizona.edu (M.S.)

**Keywords:** head and neck squamous cell carcinoma, novel therapies, human papillomavirus, immunotherapy

## Abstract

**Simple Summary:**

Human papillomavirus (HPV)-positive head and neck squamous cell carcinoma (HNSCC) represents a distinct entity with molecular, pathological, and clinical features that lead to an improved response to standard therapy and a favorable prognosis compared to HPV-negative HNSCC. In this review we expound on the developing role of immunotherapy in HPV-positive HNSCC and highlight the clinical rationale and ongoing trials investigating novel therapeutic approaches.

**Abstract:**

Immunotherapy approaches for head and neck squamous cell carcinoma (HNSCC) are rapidly advancing. Human papillomavirus (HPV) has been identified as a causative agent in a subset of oropharyngeal cancers (OPC). HPV-positive OPC comprises a distinct clinical and pathologic disease entity and has a unique immunophenotype. Immunotherapy with anti-PD1 checkpoint inhibitors has exhibited improved outcomes for patients with advanced HNSCC, irrespective of HPV status. To date, the clinical management of HPV-positive HNSCC and HPV-negative HNSCC has been identical, despite differences in the tumor antigens, immune microenvironment, and immune signatures of these two biologically distinct tumor types. Numerous clinical trials are underway to further refine the application of immunotherapy and develop new immunotherapy approaches. The aim of this review is to highlight the developing role of immunotherapy in HPV-positive HNSCC along with the clinical evidence and preclinical scientific rationale behind emerging therapeutic approaches, with emphasis on promising HPV-specific immune activators that exploit the universal presence of foreign, non-self tumor antigens.

## 1. Introduction

In the United States, head and neck squamous cell carcinoma (HNSCC) constitutes approximately 3% of all malignancies. The estimated annual incidence of HNSCC is 54,010 and HNSCC is projected to result in 10,850 deaths in 2021 [1]. Along with the known environmental risk factors, including exposure to tobacco, betel quid, and alcohol, myriad molecular and epidemiologic studies have supported the causal association between oral human papillomavirus (HPV) infection and HPV-positive oropharyngeal cancers (OPC) [2,3]. Despite a decrease in the incidence of alcohol- and tobacco-related HNSCC, coincident with declining tobacco use, the incidence of OPC has continuously risen since the 1980s. This is ascribed to an epidemic of oral HPV infection in the United States and worldwide [4,5]. While more than 220 HPV genotypes have been established, HPV16 is the predominant genotype responsible for greater than 90% of HPV-positive OPC [6,7,8].

HPV-positive HNSCC represents a distinct disease entity with molecular, pathological, and clinical features that lead to an improved response to standard therapy and a favorable prognosis compared to HPV-negative HNSCC. Weinberger and colleagues initially identified an OPC molecular profile associated with favorable prognosis: specifically, the presence of HPV16 DNA and overexpression of p16. The latter implies functionally relevant HPV; conventional thinking is that p16 is upregulated as a consequence of retinoblastoma (Rb) degradation by the oncoprotein E7 [9]. Alternately, McLaughlin-Drubin et al. have shown that p16 upregulation is Rb-independent but indeed E7-dependent, as E7 drives the upregulation of KDM6B histone demethylase [10]. Ultimately, HPV status and tobacco history were identified as the two most important prognostic factors in OPC [11], and in 2017 the American Joint Commission on Cancer codified a new staging system for p16-positive OPC to improve hazard discrimination.

Patients with recurrent/metastatic (R/M) HPV-positive HNSCC have superior survival than those with HPV-negative R/M HNSCC, despite treatment with similar regimens [4,12]. Immune checkpoint inhibitors against PD-1 have been developed agnostic to HPV status. In 2016, the anti-PD-1 monoclonal antibodies (mAb), pembrolizumab and nivolumab, were U.S. Food and Drug Administration (FDA) approved for platinum-resistant, second line, R/M HNSCC. The KEYNOTE-048 trial later demonstrated superiority of pembrolizumab versus cetuximab when combined with a platinum-5-fluorouracil doublet and administered in first line R/M HNSCC [13,14,15]. KEYNOTE-048 also introduced pembrolizumab as an appropriate monotherapy for first-line treatment in the setting of programmed cell death-ligand 1 (PD-L1) positive R/M HNSCC [14]. Despite the exciting progress in systemic therapy represented by this new class of therapeutics, for both HPV-positive and HPV-negative disease, overall survival (OS) remains dismal following failure of platinum and anti-PD-1 mAb [16,17]. 

**Figure 1:**
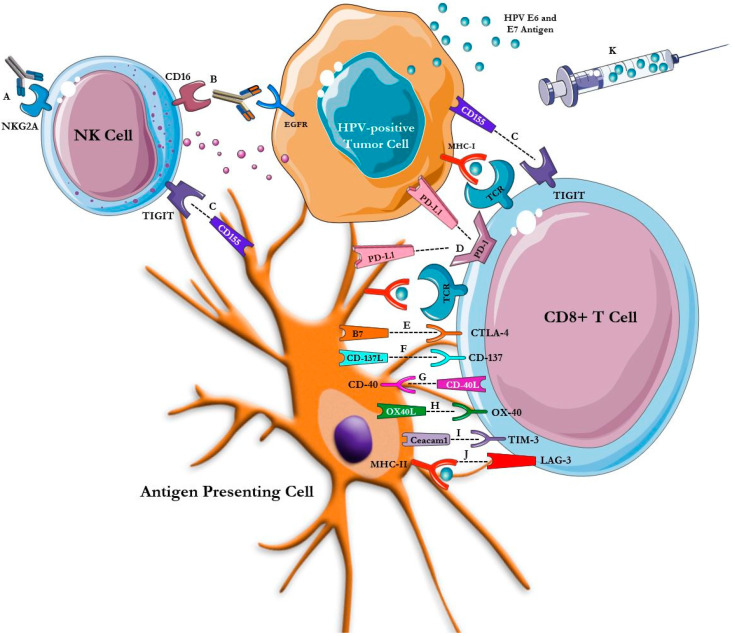
Mechanisms of Standard and Novel Immunotherapies in HPV-Positive HNSCC. The schema is a simplified illustration of the sites of action of novel immunotherapies within the tumor microenvironment of HPV-positive HNSCC. Monalizumab (A) is an anti-NKG2A mAb that augments the cytotoxic activity of NK cells. The Fc tail of cetuximab (B) and other IgG1 mAb can bind CD16 (the Fc receptor) on NK cells, triggering antibody-dependent, cell-mediated cytotoxicity. Tiragolumab (C) is a human anti-TIGIT mAb that prevents TIGIT from binding with its ligand, CD155, on dendritic and tumor cells. Nivolumab and pembrolizumab are mAb directed against the PD-1 receptor, while durvalumab and atezolizumab are mAb directed against its ligand, PD-L1 (D). Ipilimumab and tremelimumab (E) are mAb directed against CTLA-4. Urelumab and utomilumab (F) are mAb agonists of CD137. CP-870,8893 (G) is an agonist anti-CD40 mAb that activates CD40. MEDI6469 (H) is a murine-human OX40 agonist mAb. MBG453 (I) is a high-affinity, humanized IgG4 antibody targeting TIM-3. Eftilagimod alpha (J) is a is a soluble LAG-3 fusion protein that mediates antigen presenting cell activation and CD8 T-cell activation by binding to a subset of MHC class II molecules. Immunotherapeutic vaccines including MEDI0457, ISA-101, ADXS11-001 and TG4001 (K) incorporate HPV16 E6 and E7 antigens.

These poor outcomes have driven increased research into novel immunotherapeutic and targeted approaches in HNSCC. Immunotherapy has emerged as a promising therapeutic avenue in HPV-positive HNSCC as HPV-driven carcinogenesis is propelled by loss of immunologic control with chronic viral infection resulting in a unique, non-self, antigenic target [18]. In this review, we highlight the role of immunotherapy as an established standard of care, as well as explore emerging immunotherapies including novel checkpoint inhibitors, antibodies and fusion protein constructs, vaccines, and chimeric antigen receptor (CAR) T-cell therapy in HPV-positive HNSCC (Figure 1).

## 2. Primary Prevention of HPV-Positive HNSCC

HPV is a double-stranded DNA virus that infects the epidermal and mucosal surfaces of the oropharynx, genital, and anal regions and is an established causative agent in oropharynx, cervical, vulvar, anal, and penile cancers. HPV infection is common and occurs through mucosal surface contact with an infected sexual partner [19]. Three prophylactic HPV vaccinations are currently approved by the FDA including the quadrivalent HPV vaccine, Gardasil, a bivalent HPV vaccine, Cervarix, and a nine-valent vaccine, Gardasil 9, with the latter providing protection against HPV-6, 11, 16, 18, 31, 33, 45, 53, and 58 [19]. Each vaccine is recommended for a specific sex and age group. HPV vaccination for all individuals aged 9–26 years is recommended by the US Centers for Disease Control and Prevention (CDC) Advisory Committee on Immunization Practices. Additionally, the FDA has approved HPV vaccination for all individuals aged 27–45 years who have not been adequately vaccinated previously [20]. The indications for these prophylactic HPV vaccinations were established based on impact on anogenital HPV infections. However, the effectiveness against oral HPV infection specifically has only been analyzed retrospectively but was shown to have 88–93% efficacy [21]. HPV vaccinations are only effective for primary prevention with the primary mechanism of action involving the induction of anti-L1 capsid antibodies, effectively blocking the initial step of viral entry. Although numerous epidemiological studies have shown an association between oral HPV DNA detection or anti-HPV16 E6 seropositivity and consequent risk of developing OPC, no validated screening test analogous to the Papanicolaou smear exists for HPV-positive HNSCC [22]. 

## 3. Immune Microenvironment in HPV Driven HNSCC

HPV-positive OPC comprises a distinct molecular and pathologic entity with different clinical outcomes compared to HPV-negative OPCs [23]. Patients with HPV-related HNSCC are generally younger and often lack the classic risk factors including tobacco and alcohol exposure and associated medical comorbidities compared to those with HPV-negative tumors [24]. Numerous HPV genotypes cause infection, some benign and others with malignant potential. The majority of HPV-associated HNSCC in the United States can be attributed to the high-risk genotype 16, with rare accounts of HPV-18, 33, and others reported as causal [25]. High-risk HPV strain DNA can integrate into the cellular genome resulting in the subsequent overexpression of viral oncogenes E6 and E7. E6 and E7 target several intracellular signaling pathways pertinent in the cell cycle regulation, DNA repair, apoptosis, and maintenance of genomic integrity [26]. E6 binds the cell cycle regulatory protein p53 and leads to its degradation. Similarly, E7 binds the Rb tumor suppressor leading to its degradation, and consequent p16 over-expression. Together, these molecular aberrancies result in uncontrolled tumor cell growth and proliferation [27]. 

Additionally, HPV-driven HNSCC has a distinct immune microenvironment compared to non-HPV-driven HNSCC. Various mechanisms have been proposed to explain the immune evasion in these tumors including upregulation of cytokines, interference with antigen presentation, differences in immune checkpoint receptor interaction, and modulation of gene transcription [28]. HPV-positive HNSCC has increased CD8+ T-cell activation and additional markers of immune infiltration compared to HPV-negative HNSCC [29]. HPV-positive tumors also express higher levels of perforin and granzyme A and B, reflecting cytotoxic CD8+ T cell immune activation [30].

An important step in pathogenesis of HPV-driven HNSCC is escape from cell cycle regulation. Various mechanisms contribute to this aberrancy including evolution of T-cell tolerance to HPV infection, the secretion of immunosuppressive cytokines, diminutive regulation by interferon gamma, and reduced or unbalanced signal transducer and activator of transcription (STAT) 1 and 3 pathway signaling [31].

Other features unique to the immune microenvironment in HPV-driven HNSCC include increased expression of PD-L1 compared to that of HPV-negative HNSCC and normal oropharyngeal tissue. The downregulation of human leukocyte antigen (HLA) expression in HPV-driven HNSCC may also lead to decreased antigen recognition and presentation. HPV-positive HNSCC is also associated with higher expression of cytotoxic T lymphocyte-associated antigen-4 (CTLA-4), an immune checkpoint receptor, and higher regulatory T cells (Tregs) [23]. 

Investigation of 280 HNSCC tumors profiled by The Cancer Genome Atlas (TCGA) analyzed transcriptome data to comprehensively characterize the immune landscape. Notably, immune infiltration differed by HPV status: HPV associated tumors possessed increasing levels of infiltrating Tregs and CD8+ T cells, and heightened expression of the immuno-inhibitory receptor CTLA-4 compared with HPV-negative HNSCC [30]. Another TCGA study included 522 HNSCC tumors and analyzed the immune pathway-related gene expression profile using RNA sequencing. Approximately 40% of the analyzed HNSCC tumors showed an active immune signature characterized by an enhanced inflammatory response, robust cytolytic activity, and constitutive interferon-γ signaling (all, *p* < 0.001) versus an exhausted immune signature. Tumors in the oropharynx (*p* < 0.001) and HPV infection (63% versus 13% by p16 status, *p* < 0.001; 51% versus 0% by in situ hybridization status, *p* < 0.001) were characterized by this active immune signature [32]. More recently, it has been shown that HNSCC tumors that express genes predictive of T cell infiltrates (or “hot tumors”) using the TCGA portend a superior prognosis compared to tumors lacking such expression (or “cold tumors”) irrespective of tumor expression of immune inhibitory genes [33].

## 4. Immunotherapy Standard of Care Regimens in HPV-Positive HNSCC

The HPV-positive tumor microenvironment is distinct in its notable infiltration of Tregs and PD-1 positive T-cells [34]. Expression of PD-L1 and PD-L2, the ligands of the T-cell suppressive immune checkpoint receptor, PD-1, has been implicated in immune evasion resulting in recurrence or metastasis of HNSCC. Ferris and colleagues introduced the fully humanized IgG4 anti-PD-1 mAb nivolumab in CheckMate 141, an open-label, phase III trial including 361 patients with R/M HNSCC who had progressed within 6 months of receiving platinum-based chemotherapy [35]. Compared to investigator’s choice, which included single-agent chemotherapy (methotrexate, docetaxel, or cetuximab), patients who received nivolumab exhibited a significantly longer OS (hazard ratio 0.7; 97.73%CI, 0.51 to 0.96; *p* = 0.01) with limited grade 3 or 4 toxicity as compared to standard therapy (13.1% vs 35.1%). Sixty-three of 240 (26%) patients in the nivolumab cohort were HPV-positive and 29 out of 121 (24%) patients in the investigator’s choice cohort were HPV-positive. Subgroup analysis confirmed an OS benefit irrespective of HPV status, where median OS was 8.8 months in both p16-positive and -negative subjects with PD-L1 ≥ 1% versus 3.9 and 5.6 months for p16-positive and -negative subjects with PD-L1 ≥ 1%, exposed to investigator’s choice therapy). 

Two-year follow-up CheckMate 141 showed a sustained OS advantage in individuals treated with nivolumab compared to investigator’s choice, with a median OS of 7.7 vs. 5.1 months [36]. Sustained benefit in long-term (2 years) OS with nivolumab was achieved in patients irrespective of HPV status. A 40% reduction in the risk of death was noted in both the HPV-positive (HR 0.6 (95% CI 0.37–0.97)) and HPV-negative subgroups (HR 0.59 (95% CI 0.38–0.92)). An analysis stratified by both PD-L1 and HPV status found that all subgroups derived an OS benefit with nivolumab compared to investigator’s choice. However, the group positive for both HPV and PD-L1 expression sustained the highest OS benefit from nivolumab (HR 0.39 (95% CI 0.18–0.81)). 

The KEYNOTE-040 trial similarly was an international, randomized, phase III trial that demonstrated the safety and efficacy of another anti-PD-1 mAb, pembrolizumab, compared to investigator’s choice single-agent therapy including methotrexate, docetaxel or cetuximab in patients with R/M HNSCC who had progressed during or within 6 months of receiving platinum-based therapy [37]. Of the 495 patients enrolled, the median OS in the pembrolizumab cohort was 8.4 months (95% CI 6.4–9.4) vs. 6.9 months in the investigator’s cohort (95% CI 5.9–8.0). Approximately 25% and 23% of patients in the pembrolizumab and standard cohorts, respectively, had p16-positive OPC; HPV-positive subjects had similar OS benefit as the overall cohort in stratified analysis. 

More recently, KEYNOTE-048, a randomized phase III trial, evaluated the use of pembrolizumab alone, pembrolizumab plus platinum-5-fluorouracil, or the standard first-line EXTREME regimen (cetuximab plus platinum-5-fluorouracil) among 882 patients with R/M HNSCC across 200 sites and 37 countries [14]. In patients with a PD-L1 combined positive score (CPS) of ≥ 20, pembrolizumab alone showed a benefit in median OS compared to EXTREME (14.9 months vs 10.7 months, hazard ratio (HR) 0.61 (95% CI 0.45–0.83), *p* = 0.0007). In patients with CPS ≥ 1, median OS was once again superior to EXTREME (12.3 months vs 10.3 months, HR 0.78 (95% CI 0.64–0.96), *p* = 0.0086). Finally, the combination of pembrolizumab and platinum-5-fluorouracil was superior to EXTREME as first-line treatment for PD-L1 unselected, R/M HSNSCC. As KEYNOTE-048 included both HPV-positive and HPV-negative patients, with p16-positive OPC balanced across arms, the new first-line standards of care for R/M HNSCC remain agnostic to HPV status. 

## 5. Meta-Analysis of PD-1 Inhibition in HNSCC 

In an effort to further understand the impact of HPV status on response to anti-PD1 immunotherapy, recent meta-analyses have been conducted. In a meta-analysis assessing results from 11 clinical trials including 1860 patients evaluating immunotherapy in R/M HNSCC, patients with HPV-positive HNSCC demonstrated an improved risk ratio (1.29, *p* = 0.24) and OS (11.5 vs. 6.3 months) in comparison to patients with HPV-negative disease [38]. The most common immunotherapy agents across the trials were anti-PD1 agents (pembrolizumab, nivolumab) and anti-PD-L1 inhibitors (durvalumab and atezolizumab). Of note, motolimod, a Toll-Like Receptor (TLR)-8 agonist, was assessed in two trials and monalizumab, a natural killer group 2 member A (NKG2A) inhibitor, was used in one. The data also found PD-L1 to be predictive in evaluating response to immunotherapy. The best outcomes to immunotherapy were observed in patients who had tumors expressing both HPV and PD-L1. 

In another recent meta-analysis, data from 7 studies including 814 patients with R/M HNSCC were analyzed. This meta-analysis was restricted to patients treated with PD-1 or PD-L1 inhibitors as single agents. The objective response rate (ORR) of patients with HPV-positive HNSCC was significantly greater than that of their HPV-negative counterparts (OR = 1.77; 95% CI = 1.14–2.74; *p* = 0.01). The odds ratio (OR) was more impressive for the pooled anti-PD-L1 trials (OR = 2.66; 95% CI = 1.16–6.11; *p* = 0.02) in comparison to the pooled anti-PD-1 trials (OR = 1.51; 95% CI = 0.90–2.54; *p* = 0.12). Patients with HPV-positive HNSCC also were noted to have a lower risk of death in comparison to patients with HPV-negative HNSCC (HR = 0.77; 95% CI = 0.60–0.99; *p* = 0.04) [39]. 

Possible mechanisms underlying clinical benefit, including ORR and OS, in HPV-positive HNSCC treated with immunotherapy relates to the unique tumor microenvironment of HPV-positive tumors. Chiefly, the HPV-specific T-cells, type I-oriented CD4+ and CD8+ T-cells, dendritic cells, and dendritic-like macrophages, as well as the synthesis of E6 and E7 oncoproteins, induce the immune system to detect tumor cells [38]. Chakravarthy and colleagues have shown a difference in the tumor infiltrating lymphocyte levels between HPV-positive and HPV-negative OPC, reinforcing the hypothesis that a difference in immune response between the two groups may contribute to the observed survival benefit [40]. 

## 6. Novel Immune Therapies

Emerging immunotherapies in HPV-positive HNSCC include novel immune checkpoint inhibitor combinations, therapeutic vaccines, adoptive cell therapy, and gene editing approaches. Current clinical trials are summarized in Table 1.

### 6.1. Checkpoint Inhibitor Combinations with Standard Therapies 

The further elucidation of tumor immunology in the context of HPV-positive and HPV-negative HNSCC led to the hypothesis that anti-PD1 mAb may synergize with standard cytotoxic therapies, including chemotherapy and radiation, leveraging the immune response to immunogenic cell death. As observed in KEYNOTE-048, the efficacy of combining an anti-PD1 checkpoint inhibitor with cytotoxic chemotherapy displaced the then-standard of care, the EXTREME regimen. Platinum-5-fluorouracil is hypothesized increase tumor antigen presentation, immunogenic cell death, infiltration of CD8+ T-cells, and ultimately an antitumor immune response [41]. Moreover, tumor upregulation of PD-L1 expression is a consequence of chemotherapy, and is associated with worse clinical outcomes, making PD-1/PD-L1 blockade in addition to cytotoxic chemotherapy a rational therapeutic approach [42]. 

The combination of pembrolizumab plus the chimeric mouse IgG1 anti-epidermal growth factor receptor (EGFR) mAb, cetuximab, was investigated in a phase II trial in patients with platinum-resistant or -ineligible R/M HNSCC; at 6 months, the ORR was 45% (95% CI 28–62) with serious treatment-related adverse events noted in 5 of 33 (15%) participants [43]. Approximately 33% of patients enrolled in this study had HPV-positive OPC with no statistically significant disparity noted in relation to HPV status and OS or progression-free survival (PFS), although the study was not powered to detect this association.

Several clinical trials are currently underway to establish the safety and efficacy of checkpoint inhibitor combinations with curative-intent chemoradiotherapy in HNSCC. The anti-PD-L1 monoclonal antibody (mAb), durvalumab, is being assessed in combination with cetuximab and radiotherapy in locally advanced HNSCC in an ongoing phase I/II trial, including both HPV-positive and HPV-negative subjects (DUCRO; NCT03051906). A phase II/III trial in the ECOG-ACRIN oncology cooperative group is evaluating the efficacy of maintenance nivolumab after the completion of standard cisplatin chemoradiation for intermediate risk, HPV-positive OPC (EA 3161; NCT03811015). A phase II trial of ipilimumab, nivolumab, and radiation therapy in locally advanced HPV-positive OPC is currently recruiting (NCT03799445). 

A recent phase III, multicenter trial assessed the treatment outcomes of unresected locally advanced HNSCC with the addition of the PD-L1 inhibitor, avelumab, to standard cisplatin-based chemoradiotherapy versus chemoradiotherapy alone [44]. Of the 697 patients enrolled in the study, 121 (35%) of patients in the avelumab cohort were HPV-positive while 117 (34%) in the standard cohort were HPV-positive. No statistically significant difference in OS or 2-year PFS was noted between the avelumab and standard cohorts, which were stratified by HPV status. The authors hypothesized that radiation therapy depletes T cells within the tumor microenvironment, negatively impacting the capability of the immune system to control micrometastatic disease. 

### 6.2. Novel Immune Checkpoint Inhibitors 

Beyond PD-1, T-cell exhaustion markers including CTLA-4, T-cell immunoglobulin and ITIM domain (TIGIT), T cell immunoglobulin and mucin domain-containing protein 3 (TIM-3), and lymphocyte-activation gene 3 (LAG-3) have been identified as candidate targets for immune checkpoint blockade [45]. Like PD-1, these targets are upregulated in HPV-positive HNSCC compared to HPV-negative samples, likely due to the stimulus of chronic infection. Gameiro and colleagues showed a survival benefit in patients with HPV-positive HNSCC with higher expression of these exhaustion markers while no survival advantage was noted in HPV-negative HNSCC with similar elevated expression of these markers [45].

Early immune checkpoint inhibitor combinations have included anti-PD-1/L1 inhibitors with mAb targeting CTLA-4. The anti-PD-L1 mAb durvalumab was compared to investigator’s choice standard chemotherapy in the randomized phase III EAGLE trial including 736 patients with R/M HNSCC and did not improve survival as a single agent or in combination with the CTLA-4 inhibitor, tremelimumab. Although the study was not intended to evaluate OS among immunotherapies, the addition of tremelimumab did not enhance the activity of durvalumab. Moreover, HPV status did not appear to impact OS with either regimen on subgroup analysis [46]. 

CheckMate 651 is a phase III, randomized, multicenter trial studying nivolumab in combination with ipilimumab, a CTLA-4 inhibitor, versus EXTREME as first-line therapy in R/M HNSCC (NCT02741570). The trial has completed accrual; a recent press release from Bristol Myers Squibb expressed that the study did not reach its primary endpoints including OS and PFS although a positive trend was noted in OS in a subgroup of patients whose tumors expressed PD-L1 with a CPS ≥ 20 [47]. 

Novel mAb are currently being investigated in combination with PD-1/L1 inhibitors. Tiragolumab, a human anti-TIGIT mAb, is being compared to placebo in combination with atezolizumab as first-line treatment for R/M, PD-L1 positive HNSCC (SKYSCRAPER-09) (NCT04665843). Relatlimab, an anti-LAG-3 mAb, is being studied in combination with nivolumab for the treatment of R/M HNSCC (NCT04326257). 

TIM-3 is an immune checkpoint coreceptor present on activated T-cells and mediates reduced proliferation and production of effector cytokines and apoptosis of effector T-cells [48]. A recent study examined expression of TIM-3 in 80 HNSCC specimens and its correlation with clinical and pathological outcomes [48]. Increased TIM-3-positive tumor-infiltrating lymphocytes correlated with worse OS (*p* < 0.001). On multivariate cox regression analysis, high TIM-3 positive tumor infiltrating lymphocytes was noted as an independent prognostic marker for poor clinical outcome (HR 2.066 (95% CI 2.832–12.230); *p* < 0.001). Several clinical trials are underway to further investigate the safety and efficacy of TIM-3 targeting including a phase I trial evaluating TSR-022, a TIM-3 mAb in advanced solid tumors (NCT02817633). MBG453 is a high-affinity, humanized IgG4 antibody targeting TIM3 that is under investigation alone or in combination with PDR001, an anti-PD1, in patients with advanced solid malignancies including HNSCC (NCT02608268). 

### 6.3. Costimulatory T Cell Receptor Agonists 

Costimulatory receptors constitute a repertoire of second signaling molecules that propagate T cell receptor signaling, and include CD137 (4-1BB), OX40, and CD40. Sparse data exist with regards to the emerging role of costimulatory T cell receptor agonists in the context of HPV status in the treatment of HNSCC. However, in mouse models, CD137 agonists are synergistic with cisplatin and radiation therapy in inhibiting tumor growth in HPV-positive HNSCC [49]. As such, several costimulatory agonists are currently under investigation to establish their safety and therapeutic utility in HNSCC. 

Results of safety analysis of the agonist of the CD137 mAb, urelumab, which included 10 patients with HNSCC showed favorable safety profile as well as pharmacodynamic activity by inducing IFN-inducible genes and cytokines [50]. Other trials are evaluating safety and efficacy of urelumab including a phase Ib trial examining urelumab in combination with cetuximab for advanced/metastatic colorectal cancer and HNSCC (NCT02110082). In this phase Ib trial combining cetuximab with urelumab in R/M HNSCC, the expression of CD137 on tumor infiltrating lymphocytes was investigated. The trial showed enhancement of cetuximab-activated NK cell persistence, tumor antigen cross-presentation and dendritic cell maturation with the addition of urelumab [51]. The study showed that HPV-positive and HPV-negative tumors possessed a high expression of CD137 on tumor infiltrating NK cells in vivo while peripheral NK cells after cetuximab administration showed higher expressions of CD137 in HPV-positive HNSCC signifying improved outcomes of HPV-positive HNSCC treated with combination cetuximab and urelumab. Interestingly, cetuximab is an anti-EGFR, human-murine chimeric IgG1 isotype mAb and has been approved for the treatment of HNSCC in combination with chemotherapy in the R/M setting and with radiation therapy in the curative setting, although was shown to be inferior to standard cisplatin-radiation therapy in low-risk HPV-positve OPSCC [52]. In addition to EGFR signaling blockade, cetuximab has an immune mechanism of action where its IgG1 stem binds the CD16 fragment crystallizable (Fc) receptors on natural killer (NK) cells and triggers antibody-dependent cellular cytotoxicity (ADCC). In turn, the immunogenic tumor cell death caused by ADCC can result in cross-priming of CD8+ T cells with epitope spread, suggesting a possible new role for an old therapy: synergizing with novel immunotherapies [53,54].

Utomilumab (PF-05082566), also a human mAb agonist of CD137, was also studied in combination with a humanized mAb targeting CCR4, mogamulizumab, in a phase Ib study of patients with advanced solid tumors. The study demonstrated a favorable safety profile and included 11 patients with HNSCC of which 7 had disease refractory to anti-PD-1/PD-L1 therapy [55]. Utomilumab was also evaluated in combination with pembrolizumab in patients with solid tumors including HNSCC in KEYNOTE-036, a phase 1b trial that has been completed (NCT02179918). Another phase Ib/II multicenter trial, JAVELIN Medley, is evaluating the safety of avelumab in combination with utomilumab (NCT02554812).

Early phase studies include both HPV-positive and HPV-negative subjects. MEDI6469, a murine anti-human OX40 agonist antibody was studied in a phase Ib trial with 17 patients with locally advanced HNSCC prior to definitive surgical resection [56]. Two weeks after administration of MEDI6469, there was increased proliferation of CD4+ and CD8+ T cells. Tumor biopsies prior to and following surgery also confirmed an increase in CD4+ tumor infiltrating lymphocytes (NCT02274155). Another clinical trial is investigating PF-04518600, an OX40 agonist, alone or in combination with the 4-1BB (CD137) agonist PF-05082566 (NCT02315066) in patients with select advanced or metastatic carcinoma including HNSCC. 

The CD40 ligand is present on antigen presenting cells and serves as a key mediator for cellular and humoral immune response. Activation of CD40 results in augmented antigen presentation and apoptosis induction [57]. CP-870,8893, an agonistic anti-CD40 mAb that can activate CD40, is under study in a phase I trial in patients with advanced solid tumors (NCT02225002). A favorable safety profile and biological and clinical response has been identified with the use of CP-870,8893 coupled with carboplatin and paclitaxel for patients with advanced solid tumors [57].

### 6.4. Novel Antibodies and Fusion Protein Constructs 

Reinforcing the innate immune system to mediate anti-tumor activity by augmenting NK cell antibody-dependent cell-mediated cytotoxicity is a strategy that is increasingly under consideration [58]. Monalizumab, a mAb targeting NKG2A, is currently under investigation in HNSCC [58,59]. The NKG2A heterodimeric receptor is a prominent NK inhibitory receptor. Monalizumab prevents binding of the HLA E ligand, often overexpressed in HNSCC, to NKG2A resulting in increased cytotoxic activity [58]. The relevance of HPV status for the activity of monalizumab is currently unknown. In a Phase 1b/2 trial presented at the European Society for Medical Oncology (ESMO) 2019 congress, the combination of monalizumab 10 mg/kg every 2 weeks and cetuximab showed an acceptable safety profile and a response rate of 27.5% (36% and 17% in anti-PD1 immunotherapy-naïve, *n* = 22, and immunotherapy-pretreated patients, *n* = 18, respectively) in 40 patients who had progressed after platinum-based chemotherapy and had received ≤2 prior lines of therapy [60]. The Phase II expansion cohort evaluating monalizumab and cetuximab in immunotherapy-pretreated patients affirmed an ORR of 20% across 40 patients enrolled [61]. A phase III clinical trial of monalizumab plus cetuximab compared to placebo plus cetuximab is currently underway in R/M HPV-positive and negative HNSCC following progression on standard platinum and anti-PD1 immunotherapy (NCT04590963).

The presence of HPV16 oncoproteins, natural non-self antigens, in HPV-positive HNSCC raises the promise of developing HPV-specific immunotherapies. CUE-101, a novel fusion protein construct of the major histocompatibility complex (MHC) HLA-A*0201 allele bound to an epitope from the HPV 16 E7 protein, is currently being studied in a phase I clinical trial. CUE-101 was developed to activate and expand tumor-specific T cells targeting HPV16-driven cancers. In preclinical murine models, selective binding and preferential activation and expansion of E7-specific T cells was achieved, with dose-dependent cytokine production, inhibition of tumor growth and increasing survival both as a monotherapy and in combination with an anti-PD-1 antibody [62]. CUE-101 is currently under study in a phase I trial both as monotherapy in second line or later HPV-positive HNSCC and in dose-escalation and expansion in combination with pembrolizumab in the front-line metastatic stetting (NCT03978689). 

Eftilagimod alpha (IMP-321) is a soluble LAG-3 fusion protein that mediates antigen presenting cell activation and CD8 T-cell activation by binding to a subset of MHC class II molecules. A phase II study (TACTI-002) evaluated the safety and efficacy of eftilagimod and pembrolizumab for second-line treatment of advanced HNSCC and non-small cell lung cancer. All 18 subjects with HNSCC were noted to have immune partial response. The study concluded safe and effective antitumor activity in the second-line setting for R/M HNSCC [63]. Eftilagimod Alpha is under phase II evaluation in combination with pembrolizumab for patients with R/M HNSCC (NCT03625323). 

### 6.5. Vaccines

HPV-positive HNSCC contains promising non-self antigens, including E6 and E7, which are specific to HPV-infected cells and not healthy cells. The E6 and E7 oncoproteins are requisite for maintenance of the aberrant phenotype, and as foreign antigens will be recognized by the human immune system [64]. Enhancing anti-HPV T cell activity is the goal of several HPV-targeted therapeutic vaccines currently under investigation in HPV-positive HNSCC. 

MEDI0457 is a DNA immunotherapeutic vaccine of HPV-16 and HPV-18 E6/E7 expressing plasmids coupled with IL-12 expressing plasmids [65]. In a phase Ib/II pilot study consisting of 21 patients with locally advanced, p16-positive HNSCC, MEDI0457 was dosed perioperatively or following completion of concurrent chemoradiotherapy. An increase in the tumor-infiltrating CD8+ T-cell: regulatory T-cell ratio was noted following MEDI0457 administration as was the presence of HPV-16-specific T-cells [65]. MEDI0457 was studied in combination with anti-PDL1 durvalumab in a phase Ib/IIa trial in patients with HPV-positive R/M HNSCC who had progressed on at least one prior regimen [66]. At interim analysis, ORR was 22.2% with 3 complete responses (CR) and 3 partial responses (PR). Treatment-related adverse events (AE) were noted in 77.1% of patients, predominantly grade 1–2. Fatigue (37.1%) and injection site pain (34.3%) were the most common AEs. No patients had a grade 4/5 treatment-related AE. An increase in both tumoral CD8+ T cells as well as peripheral HPV-specific T cells was shown. 

ISA-101 is an HPV-16 vaccine comprised of HPV-E6 and E7 long peptides. These long peptides include cytotoxic-T lymphocyte epitopes and T-helper epitopes in order to effectively stimulate HPV-16-specific responses [67]. ISA-101 produced strong immune responses to HPV-16, but vaccination alone was not effective for invasive cancer treatment. A phase II trial further looked at the combination of ISA-101 and checkpoint inhibition with nivolumab, a PD-1 antibody, in patients in R/M HPV-16-positive cancers [68]. The ORR was 33%. The majority of patients, 22 of 24, had advanced OPC; among this cohort, the PR rate was 27% while the CR rate was 9%, and the disease control rate was 45%. The most prevalent AEs in the study encompassed injection site reactions, fever, diarrhea, and hepatotoxicity. ISA-101 is also under investigation with utomilumab, a CD-137 agonist, for patients with incurable HPV-16-positive OPC (NCT03258008) [69]. ISA-101 is also being studied in a phase II trial in combination with pembrolizumab and cisplatin-based chemoradiation for patients with newly diagnosed, locoregionally advanced, intermediate risk HPV-associated HNSCC (NCT04369937). 

ADXS11-001 is a live-attenuated Listeria monocytogenes (LM) vaccine encoding a HPV16 E7 oncoprotein interconnected to LM listeriolysin O and is being evaluated in a neoadjuvant window of opportunity trial prior to robot-assisted resection of HPV-positive OPC. The co-primary endpoints are HPV-specific T cell response rate and safety. Preliminary results included a high rate (56%) of serious adverse events including one death (NCT02002182).

A modified vaccine virus Ankara (MVA) vector expressing HPV16 E6/E7 and IL-2, TG4001 (tipapkinogene sovacivec), was evaluated in combination with avelumab in patients with R/M HPV-16+ cancers in a phase 1b/2 trial [70]. At interim analysis, responses were noted with no dose-limiting toxicities or serious adverse events. TG4001 with avelumab was also shown to induce a shift from a “cold” tumor to a “hot” tumor gene signature with an increase of CD8 infiltration, a decrease in infiltrated Treg/CD8 ratio as well as an increase the proportion of PD-L1+ cells in low to moderate PD-L1 expressing tumors.

PRGN-2009 is a novel HPV adenovirus vaccine that includes multiple E6/E7 epitopes of HPV16 and HPV18. It is being evaluated in a phase I/II clinical trial alone or in combination with anti-PD-L1/TGFbeta trap (M7824) in patients with advanced or metastatic HPV+ malignancies including HPV+ OPC (NCT04432597). 

AMV002, a therapeutic HPV DNA vaccine, has been investigated in a Phase I study for patients with prior treatment for HPV-positive OPSCC to evaluate safety and immunogenicity [71]. It was deemed to be tolerated at all dose levels and enhanced immunity to virus-derived tumor-specific antigens.

DPX-E7 is a synthetic peptide-based vaccine of HPV16 E711-19 currently being investigated in an open-label phase Ib/II trial including HLA-A*02-01 patients with HPV16-associated head and neck, anal, and cervical cancers (NCT02865135).

### 6.6. Adoptive T Cell Transfer 

In adoptive cell-based therapies, dendritic cells, B cells and/or T cells are isolated from a patient, transduced ex vivo in order to express or target a specific antigen, then infused back to the same patient [72,73]. CAR-T cells are engineered T cells that express a single-chain fragment variable, which can in turn recognize a tumor-specific antigen. CD3-related components and costimulatory domains are also often included. MHC class I molecules process and present epitopes from E6 and E7 and may be optimal targets for T cell receptor (TCR) gene engineered T cells [73]. An E6-specific anti-PD1 engineered TCR T cell is under evaluation in a phase I study for patients with HPV-positive HNSCC or cervical cancer (NCT03578406). An E7 TCR-engineered T cell is being investigated in a phase I/II clinical trial in HPV16-associated cancers including OPCs (NCT02858310). 

## 7. Conclusions

The field of immuno-oncology has progressed rapidly. This new treatment paradigm has emerged with better understanding of the interaction between the tumor, tumor microenvironment, and the immune system. Recent advances in immune checkpoint inhibition have positively changed the outcomes in both HPV-positive and HPV-negative HNSCC. While representing a major advance, first generation anti-PD1 mAb are non-specific immunotherapies and do not leverage the tumor antigen specificity represented by HPV infection. Progressive insight into the tumor microenvironment of HPV-positive HNSCC, including the unique interplay between chronic foreign antigen stimulation by HPV oncoproteins and T cell exhaustion, is inspiring new immunotherapeutic agents and combinations. Most promising, the advent of HPV-specific immune activators, such as novel fusion proteins, anti-HPV therapeutic vaccinations, and HPV-specific adoptive T cells that exploit the universal presence of foreign viral antigens, blazes a trail toward precision immunotherapy.

## Figures and Tables

**Table 1:** Clinical trials evaluating novel immunotherapies in HPV-positive HNSCC.

Treatment	Indication	Clinical Trial ID
Anti-PD-1/L1 Checkpoint Inhibitor Plus Standard of Care Combinations		
Durvalumab (anti-PD-L1), cetuximab (anti-EGFR), and radiation therapy	Locally advanced HNSCC	NCT03051906
Ipilimumab (anti-CTLA4), nivolumab (anti-PD1), and radiation therapy	HPV-positive advanced OPCs	NCT03799445
Maintenance cemiplimab (anti-PD1)	Locally advanced HNSCC	NCT04831450
Nivolumab plus ipilimumab compared to the standard of care (EXTREME Regimen)	First-line R/M HNSCC	NCT02741570
Nivolumab/carboplatin/paclitaxel	HPV-positive OPC	NCT03342911
Nivolumab plus paclitaxel	R/M HNSCC	NCT04282109
**Novel Immune Checkpoint Inhibitors**		
Atezolizumab (anti-PD-L1) plus tiragolumab (anti-TIGIT)	R/M PD-L1 positive HNSCC	NCT04665843
Nivolumab plus relatlimab (anti-LAG-3)	R/M HNSCC progressed on prior immunotherapy	NCT04326257
TSR-022 (anti-TIM-3)	Advanced solid tumors	NCT02817633
MBG453 (anti-TIM-3) ± PDR001 (anti-PD1)	Advanced solid tumors	NCT02608268
**T Cell Receptor Costimulatory Agonists**		
Anti-OX40 Antibody (OX40 agonist)	Head and neck cancers	NCT02274155
PF-04518600 (OX40 agonist) alone/or in combination with PF-05082566 (4-1BB agonist)	Advanced or metastatic carcinoma	NCT02315066
PF-05082566 plus pembrolizumab (anti-PD1)	Advanced solid tumors	NCT02179918
Avelumab (anti-PD-L1)in combination with utomilumab (4-1BB agonist)	locally advanced or metastatic solid tumors	NCT02554812
Urelumab (4-1BB agonist) and cetuximab	Advanced/metastatic head and neck cancers	NCT02110082
CP-870,893 (CD40 agonist)	Advanced solid tumors	NCT02225002
**Novel Antibodies and Fusion Proteins**		
Monalizumab (NK cell NKG2A inhibitor) plus cetuximab	R/M HNSCC	NCT04590963
CUE-101 (HPV-16 E7 T cell activator)	HPV-positive R/M HNSCC	NCT03978689
Eftilagimod alpha (soluble LAG3 protein) and pembrolizumab	R/M HNSCC	NCT03625323
PDS0101 (liposomal multipeptide vaccine targeting HPV-16 E6 and E7) + NHS-IL12 (Interleukin-12) + M7824 (bifunctional fusion protein targeting TGF-β and PD-L1)	HPV-positive cancers	NCT04287868
**Vaccines**		
Utomilumab and ISA101b vaccine (synthetic long HPV16 E6/E7 peptides vaccine)	HPV-positive OPCs	NCT03258008
ISA101b and pembrolizumab plus cisplatin	HPV-positive HNSCC	NCT04369937
ADX 11-001 Vaccine (live attenuated Listeria monocytogenes bacterium)	HPV-positive OPC	NCT02002182
PRGN-2009 Vaccine (novel gorilla adenovirus vaccine) alone or in combination with M7824 (anti-PDL1/TGF-beta trap)	HPV-positive cancers	NCT04432597
DPX-E7 Vaccine (synthetic peptide-based vaccine of HPV16 E711-19)	HPV-positive OPC, cervical and anal cancers	NCT02865135
BNT113 Vaccine (RNA-lipoplex (RNA-LIP)-based mRNA vaccine encoding HPV-16 E6 and E7) plus pembrolizumab	HPV-positive and PD-L1 expressing HNSCC	NCT04534205
PDS0101 plus pembrolizumab	HPV-positive HNSCC	NCT04260126
**Adoptive T-cell Transfer**		
RPTR-168 (Autologous IL-12/multi-targeted primed T cells)	HPV-positive HNSCC, cervical and melanoma	NCT04762225
E7 TCR T cells	HPV-positive cancers	NCT02858310
T Cell Receptor Immunotherapy Targeting HPV-16 E6	HPV-positive cancers	NCT02280811

Abbreviations: CAR, chimeric antigen receptor; EXTREME indicates cetuximab/platinum/5-fluorouracil; HPV, human papillomavirus; HNSCC, head and neck squamous cell carcinoma; IL, interleukin; OPC, 1oropharyngeal cancer; PD-1, programmed cell death protein 1; R/M, recurrent/metastatic.
